# PD-L1 biology in response to chemotherapy in vitro and in vivo in ovarian cancer

**DOI:** 10.1186/2051-1426-3-S2-P302

**Published:** 2015-11-04

**Authors:** Shannon Grabosch, Feitianzhi Zeng, Lixin Zhang, Mary Strange, Joan Brozick, Robert P Edwards, Anda Vlad

**Affiliations:** 1 Magee-Womens Hospital of UPMC, Pittsburgh, PA, USA; 2 Magee-Womens Research Institute, Pittsburgh, PA, USA; 3 Magee-Womens Research Institute Ovarian Cancer Center of Excellence, Pittsburgh, PA, USA

## Objective

PD-L1 is an immune checkpoint molecule expressed by a variety of tumors, including ovarian, which binds to circulating PD-1 expressing effector T cells allowing for tumor escape from the immune system. PD-L1 blockade prevents PD-L1/PD-1 interaction and is currently explored as therapy of solid tumors. Ovarian cancer patients receive combination cisplatin/taxane chemotherapy as standard of care. Chemo-induced effects on tumor PD-L1 expression have been only partially addressed. We studied here the effect of platinum/taxane exposure on PD-L1 expression in vitro and in vivo.

## Methods

Human (OVCA 420 and OVCA432) and mouse (2F8) ovarian cancer cell lines were exposed to increasing doses of cisplatin and paclitaxel for different time periods. PD-L1 expression was analyzed with flow cytometry and Western blot. Through continuous exposure in vitro of mouse 2F8 ovarian cancer cells to increasing doses of cisplatin we have derived a new cisplatin-resistant line (2F8-Cis). In vivo, we have challenged n=37 mice IP with 0.8 million 2F8 cells. Tumor-bearing mice were treated with cisplatin, anti-PD-L1 antibody, both drugs, or isotype control every two weeks for three doses starting at day 14 post-inoculation. Tumor- and ascites-derived cancer cells were analyzed with flow cytometry.

## Result

Exposure of OVCA420 and OVCA432 to cytotoxic doses of cisplatin or paclitaxel trigger PD-L1 up-regulation. Similarly, 2F8-Cis cells show increased cell surface PD-L1 compared to parental 2F8 cells, providing the rationale for combination therapy with PD-L1 blockade. In vivo treatment of mice with aggressive 2F8 tumors respond well to cisplatin and anti-PD-L1 individually with increased survival (median 45 days versus 24 days for isotype control, p=0011). At necropsy, anti-PD-L1 therapy significantly reduced tumor burden (1.48 g versus 0.25 g, p=0.0294). Tumor cells cultured from cisplatin-only treated mice expressed higher levels of PD-L1, in line with our in vitro results. A higher percentage of PD-1 expressing cells were found amongst the tumor cells in these cultures versus cisplatin/anti-PD-L1 treated mice. Although high dose anti-PD-L1 immediately following cisplatin administration can control tumor burden (0.48 g), it does not significantly prolong survival (median 29 days). We are currently testing an alternative therapeutic schema exploring a lower anti-PD-L1 dose and a different timing post-chemo.

## Conclusion

Tumor cells upregulate PD-L1 in response to chemotherapy exposure and combination PD-L1 blockade in conjunction with chemotherapy effectively controls tumor burden. Optimization of timing and dosage for this combination therapy will likely increase its therapeutic benefit.

